# Erratum for Euro Surveill. 2015;20(45)

**DOI:** 10.2807/1560-7917.ES.2016.21.38.30344

**Published:** 2016-09-22

**Authors:** Eurosurveillance editorial team

**Affiliations:** 1 European Centre for Disease Prevention and Control (ECDC), Stockholm, Sweden

In the article ‘Carbapenemase-producing *Enterobacteriaceae* in Europe: assessment by national experts from 38 countries, May 2015' by B Albiger et al., published on 12 November 2015, Table 4 was corrected and replaced on 22 September 2016.

